# Scaling in branch thickness and the fractal aesthetic of trees

**DOI:** 10.1093/pnasnexus/pgaf003

**Published:** 2025-02-11

**Authors:** Jingyi Gao, Mitchell G Newberry

**Affiliations:** Department of Computer Science, University of Wisconsin, Madison, WI 53706, USA; Department of Biology, University of New Mexico, Albuquerque, NM 87131, USA; Center for the Study of Complex Systems, University of Michigan, Ann Arbor, MI 48109, USA

## Abstract

Leonardo da Vinci left guidelines for painting trees that have inspired landscape painters and tree physiologists alike, yet his prescriptions depend on a parameter, *α*, now known as the radius scaling exponent in self-similar branching. While da Vinci seems to imply α=2, contemporary vascular biology considers other exponents such as the case of α=3 known as Murray’s Law. Here we extend da Vinci’s theory of proportion to measure *α* in works of art, enabling comparison to modern tree physiology and fractal geometry. We explain how *α* determines proportions among branches and visual complexity, which in turn influence the fractal dimension *D*. We measure *α* in classic works of art drawn from 16th century Islamic architecture, Edo period Japanese painting and 20th century abstract art. We find *α* in the range 1.5 to 2.8 corresponding to the range of natural trees, as well as conformity and deviations from ideal branching that create stylistic effect or accommodate design and implementation constraints. Piet Mondrian’s cubist abstract *Gray Tree* furthermore foregoes explicit branching but conforms to the theoretically predicted distribution of branch thickness with α=2.8, suggesting that realistic scaling is as important as branching in conveying the form of a tree.

**Significance Statement**

Viewers across centuries have marveled at artwork depicting trees and other fractal patterns. We analyze trees in artwork as self-similar, fractal forms, and empirically compare art with theories of branch thickness developed in biology. The key parameter *α* is analogous to the fractal dimension, but while fractal dimension varies considerably across trees and artwork, we find that the range of *α* in case studies of great artworks across cultures and time periods corresponds to the range of real trees. We relate *α* to proportions among branches, overall visual complexity, and hydraulic and mechanical constraints on trees. Finally, abstract paintings with realistic *α* are recognizable as trees, whereas an otherwise similar painting is no longer distinctly recognizable as a tree.

## Introduction

Classical theories of proportion, such as the rule of thirds or golden sections, describe ratios between particular dimensions in a design. Vitruvius, for example, laid out proportions in architectural embellishments by analogy with the human form, as famously iconified in da Vinci’s Vitruvian Man. Yet trees do not conform to simple prescribed ratios. Each branch creates smaller versions of itself, altering their proportions in response to subtleties of light, wind, temperature, and gravity.

Rendering trees thus requires more flexible or recursive rules, such as da Vinci’s area preserving rule (1, 2) or L-systems (3). Artistic and scientific idealizations of trees highlight different aspects yet share common mathematical descriptions, and so the art of trees has value even in biology, where measurement can be difficult due to damage, shade, bark, and other complexities of the real world (4, 5).

Idealized trees and many other recursive or self-similar forms are now understood to be fractals. Fractal geometry describes shapes with ever-increasing detail and variation at finer and finer scales using the concept of statistical self-similarity: objects composed of smaller random renditions of themselves. Trees and fern fronds are now textbook examples of self-similar fractals (3, 6).

Intrigue has surrounded fractals as aesthetic objects per se since their earliest computer renderings produced arresting images reminiscent of natural objects but never before seen in nature (7). Fractal patterns have further appeared in art and have long held aesthetic and spiritual value across cultures. Traditional African and Indian art, architecture, and cosmology are replete with fractal shapes, self-similarity, and self-reference (8, 9), while ancient Chinese calligraphy critics prized script that ineffably resembled spectacular summer clouds, the cracks in a wall and water stains of a leaking house—each prototypical fractals (10).

Yet relating aesthetic value to properties of fractals such as their definitive property—the fractal dimension—has proved challenging. Psychological studies have found some fractal shapes pleasing or reassuring (11, 12) and viewers sometimes prefer intermediate fractal dimension within sets of images (13). However, pleasing and displeasing forms exist in fractal and nonfractal patterns (14) and preferences for fractal dimensions are both context-dependent (15) and subjective (16). Despite considerable investigation into the fractal dimension of drip paintings by Jackson Pollock (17), claims regarding aesthetics, uniqueness to Pollock, and even fractality have been disputed (18–20). The very ubiquity of fractals in nature also makes it difficult to disentangle fractality as causative or consequential of aesthetic merit.

Trees offer a revealing case in which fractal properties have a physical and biological basis as well as aesthetic quality. Theories of optimal transport networks predict pervasive fractal structure across living things, as the outcome of convergent evolution towards maximizing exchange surface capacity for metabolism while minimizing material transport costs (21, 22). Such theories predict scaling relationships—descriptions of how properties change with size or scale of observation. For example, optimizing the energy efficiency of fluid flow gives a formula for a single fluid-conducting vessel, known as Murray’s Law (23), while scaling relationships extrapolate from microscopic rules to whole-system properties such as metabolic efficiency (24) and total leaf area (25). Scaling relationships can also combine to predict fractal dimension (21, 24), which itself is defined in terms of a scaling relationship (26).

Tree branching involves fluid transport (4) as well as light harvesting (27) and mechanical stability (2). These sometimes conflicting demands imply different optimal scaling relationships, and plants make tradeoffs that manifest in variation from individual to individual and species to species. Some vines, for example, scale according to Murray’s Law, optimizing fluid transport efficiency and neglecting mechanical support altogether, whereas woody trees grow thicker trunks and branches for mechanical support, resulting in scaling closer to da Vinci’s Law (28).

Here we study scaling of branch thickness in artistic depictions of trees. Starting from da Vinci’s observations of tree growth, we derive mathematical rules for proportions among branch diameters and for the approximate number of branches of different diameters, relying only on basic geometry and algebra. We provide formulas, code, and examples, as guidelines for painting and improvements to algorithmic generation of trees.

We write formulas leaving the branch radius scaling exponent *α* as a free parameter. We then use inferential statistics developed for biology to measure *α* in classic works of art chosen to represent pleasing trees in diverse contexts. We find that the range of *α* in great art resembles the range for real trees. Psychologists, physicists, and art historians have struggled to reliably associate aesthetic quality with particular values of fractal dimension *D* (14–16). Yet *α* implies a system of proportions involved in both visual aesthetics and the physiology of trees, and associates the physiological range of real trees with time-honored works of art that transcend artistic style, time period, and culture of origin. We therefore put forth *α* as a potentially more informative measure for studying empirical relationships between fractals and aesthetics in trees.

Finally, we argue that natural scaling in branch diameter cues viewers to perceive trees. We examine increasingly abstract paintings by Piet Mondrian (1872–1944) as a natural experiment. Mondrian’s *Gray Tree* (1911) clearly depicts a tree, but uses only black arcs on a gray background without branching, leaves, color, or other obvious visual cues. The painting distills a tree into only its most essential visual elements, yet we show that among these essential elements is realistic scaling of branch thickness. As if to test the point, Mondrian’s subsequent painting, *Blooming Apple Tree* (1912) removes even scaling in branch diameter, and the effect of a tree disappears. Without the natural variation in branch diameter, *Blooming Apple Tree* reads equally as dancers, fish, flowers, or simply a nonrepresentational abstract form. By successively removing elements until nothing left can be taken away, Mondrian’s tree paintings show that scaling in branch diameter is essential to trees.

### Proportion and the physics of trees

**Fig. 1.**
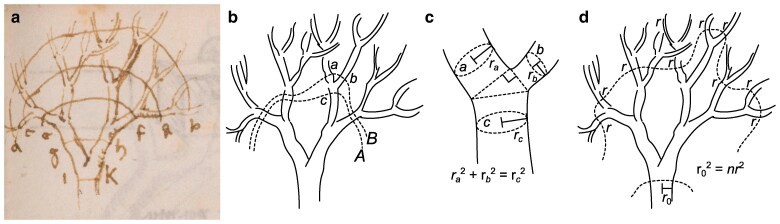
a) da Vinci’s sketch of a tree illustrates the principle that combined thickness is preserved at different stages of ramification. b) Transects *A* and *B* have the same combined thickness and so limbs *a* and *b* together must have the same thickness as *c*. This has been taken to imply that c) combined cross-sectional area ($\pi r^{2}$) is preserved across branching. Likewise, d) such transects can count the number of branches *n* that have radius approximately *r* to derive a fractal scaling relationship. Source: (a) from Institut de France Manuscript M, p. 78v.

Da Vinci’s 1495–1500 Manuscript M describes a general and flexible rule for the thickness of tree branches (1),

Every year when the boughs of a tree have made an end of maturing their growth, they will have made, when put together, a thickness equal to that of the main stem; and at every stage of its ramification you will find the thickness of the said main stem; as: *i k*, *g h*, *e f*, *c d*, *a b*, will always be equal to each other,

accompanied by a diagram reproduced in Fig. 1a. Scholars have interpreted “thickness” as the cross-sectional area of a branch, although we will show that other interpretations are reasonable and correspond to different values of *α*.

Da Vinci’s picture readily implies two equations, one describing the proportions among adjoining boughs, and one describing the number of branches of a given size. The first equation states that combined cross-sectional area is preserved wherever one bough branches into two:


(1) $$r_{a}^{2}+r_{b}^{2}=r_{c}^{2}.$$

Here $r_{a}$ and $r_{b}$ are the radii of any two adjoining downstream (distal) branches *a* and *b* and $r_{c}$ is the radius of the upstream (proximal) branch *c*, as in Fig. 1c. As proof, observe that we can choose two transects *A* and *B* as in Fig. 1b that bisect the tree (separating the leaves from the roots exactly once) and differ only in whether they incorporate *a* and *b* versus *c*. The area is $\pi r^{2}$ for each branch with radius *r*, and so equating the combined area of the two transects gives $\pi r_{a}^{2}+\pi r_{b}^{2}+\cdots =\pi r_{c}^{2}+\cdots$ where “⋯” represents branches shared by both transects. The shared branches and factors of *π* cancel from both sides. The resulting equation (Eq. 1) as well as the exponent, 2, are known in tree physiology as da Vinci’s Rule (2).

The second equation counts branches with a certain approximate thickness. Again, we choose transects to bisect the tree in two different ways as in Fig. 1d, this time to count the number *n* of branches with radius approximately *r*. These transects give the equation $n\pi r^{2}\approx \pi r_{0}^{2}$, because *n* cross-sections with area approximately $\pi r^{2}$ must sum to equal the main stem’s area $\pi r_{0}^{2}$. We solve for *n* to reach


(2) $$n\approx {\left(\frac{r_{0}}{r}\right)}^{2}.$$

This proof is exact for ideal, symmetric trees (cf. (29)), but inexact for realistic trees with random asymmetry among branches, where no branch radius is exactly *r*. Eq. 2 is nonetheless simple, general and reliable to good approximation.

Furthermore, Eq. 2 is a scaling relationship akin to the scaling relationship underlying the concept of fractal dimension (7). Mandelbrot famously described fractals according to how much more detail exists when we sharpen the clarity of observation (26). If we choose *r* to be the smallest observable branch radius in a photograph, the number of branches *n* counts the details at the finest scale. If we then increase the camera resolution *x*-fold, branches of size r/x will now be visible, revealing $x^{2}$ times more of them.

Modern science does not take da Vinci’s Rule or the exponent 2 as absolute, but rather as one point on a spectrum (5, 30). Da Vinci identifies cross-sectional area with fluid flow due to conservation of mass: “all the branches of a watercourse at every stage of its course, if they are of equal rapidity, are equal to the body of the main stream.” Yet scientists no longer believe that the water in trunks and twigs flows with equal rapidity (4), nor that the diameter of a tree branch is determined only by its water-carrying capacity (2, 28). Regarding the first point, Cecil Murray in the early 20th century reasoned that evolution by natural selection should optimize vessel diameter so that the flow rate is proportional to $r^{3}$ rather than cross-sectional area $r^{2}$, using a generic fluid dynamic argument that applies to blood vessels as well as the water conduits in the xylem of trees (4, 23). Murray’s $r^{3}$ requires combined cross-sectional area to increase to accommodate a greater volume of slower-moving water and still equal the same total flow. Fluid flow within trees is still an active research topic (31). Regarding the second point, woody tree branches serve multiple functions, such as supporting the weight and wind load of leaves in addition to conducting water. Mechanical resilience implies different scaling relationships for branch thickness (28), and different trees adopt different tradeoffs that may change with their lifetime (5), environment, diseases or mechanical damage (32).

Fortunately, our earlier arguments underlying Eqs. 1 and 2 justify simply replacing $r^{2}$ with $r^{\alpha }$. We postulate that fluid flow is governed by an unknown equation of the form $f=cr^{\alpha }$ instead of $a=\pi r^{2}$ and carry the arguments through as before, assuming that combined fluid flow must be equal at every level of ramification rather than combined area. This assumption is equivalent to conservation of mass, and fluid flow is a good proxy for many physiological quantities in trees such as rate of photosynthesis, respiration and total downstream leaf area. The form $f=cr^{\alpha }$ is reasonable because physical formulas such as Murray’s Law result in equations of this form according to dimensional analysis, provided that the laws of physics and the prevailing mechanisms are the same at all scales. In deference to the mysteries of trees, then, we leave the constant *c* and the exponent *α* as unknowns. The constant *c* immediately disappears from all equations just as *π* disappears from Eqs. 1 and 2, as does, incidentally, any conversion factors between units or radius versus diameter. Only the exponent *α* remains as a central free parameter. The result is generalized versions of the equations:


(3) $$r_{a}^{\alpha }+r_{b}^{\alpha }=r_{c}^{\alpha }$$

and


(4) $$n={\left(\frac{r_{0}}{r}\right)}^{\alpha }.$$

Recapitulation of the arguments in terms of *f* clarifies that the radius scaling relationship and the power of radius preserved across ramification have one and the same exponent, *α*, originating from the phenomenological relationship between fluid flow and branch radius.

The flow rate *f* also corresponds to the “thickness” which makes the stages of ramification equal to the main stem. Thickness (*grossezza* or “size” in the original) might reasonably refer to diameter (proportional to *r*), area (proportional to $r^{2}$) or some other measure of size, but da Vinci’s statement can only be true for one of these alternative interpretations. Leaving *α* as an unknown in $f=cr^{\alpha }$ lets $r^{\alpha }$ represent the measure of size that makes da Vinci’s statement true—be it diameter, area, $r^{3}$ or something in between—and opens the possibility of inferring *α* from data.

### Aesthetics of *α*

**Fig. 2.**
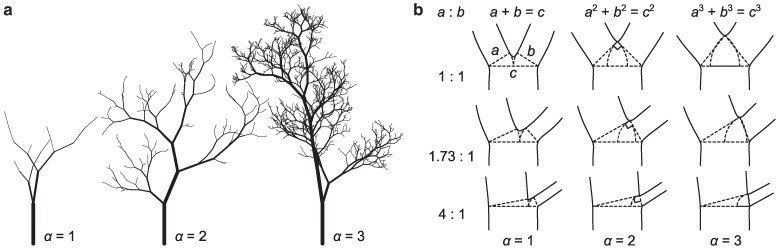
The scaling exponent *α* (a) determines the amount of detail at a given resolution and (b) proportions among adjoining boughs. a) Three trees generated by the same random algorithm differ only in the value of *α* used to compute the branch diameters. The algorithm stops adding branches when the radius reaches roughly 1/10th that of the trunk, creating exponentially more branches for higher values of *α*. Implementation of the algorithm in JavaScript and SVG is described in Methods and included in the accompanying git repository. b) *α* constrains the proportions among adjoining branches. Diameters of the downstream boughs *a* and *b* relate to the upstream bough diameter *c* as $a^{\alpha }+b^{\alpha }=c^{\alpha }$. The proportion a:b as well as *α* are required for Eq. 3 to uniquely determine the proportions among all three branches.

The exponent *α* influences multiple visual aspects of trees—both proportions among branches and the total amount of detail in a figure. Small differences in the scaling exponent create stark differences in the total amount of detail. Figure 2a depicts three trees, each with a 10-fold contrast in diameter between the largest and smallest branches. Setting α=1 leads to a rapid reduction in branch thickness and relatively few branches. Taking α=3 allows a gradual reduction in branch size and hence more ramifications within the same size range. Each ramification in turn leads to twice as many downstream branches, and hence increasing *α* produces exponentially more detail.

The influence of *α* on the proportions among adjoining branches is more subtle, as shown in Fig. 2b. As a single equation with three unknowns, Eq. 3 does not specify the proportions between branches, but rather a spectrum of possible proportions at a given value of *α*. The proportions are exactly specified given *α* along with the ratio of a:b. With α=1 or α=2, these relationships can be visualized easily. In the first case, the diameters *a* and *b* simply sum to *c* as in the first column of Fig. 2b. For α=2, the possible relationships between diameters are the same as the sides of a right triangle, because in that case Eq. 3 corresponds to the Pythagorean Theorem. For α=3, however, there is no such visual aid. The classical problem of “doubling the cube”—equivalent to the simplest case of the symmetric ratios in the top left of Fig. 2b—was attempted since antiquity and later proven to be impossible to solve with only straightedge and compass geometry (33). Solutions to cubic ratios therefore resort to less visual means—algebra and computation.

The proportions implied by α=1 versus 2 differ substantially enough to a painterly eye for da Vinci to remark on the constraint and argue for 2. Yet the exponents 2 and 3 are barely distinguishable in the second and third columns of Fig. 2b without aid from geometry, and accessing exact proportions for α=3 requires computation. Nonetheless, these subtle differences compound exponentially over successive stages of branching to create striking differences in the amount of detail in a rendering.

In simple figures of trees with relatively few branches, the value of *α* matters for determining the proportions among adjoining boughs. In this case, proportioning branches according to α=2 provides an easy, visually accessible rule that is often good enough. With more successive ramifications or more contrast in size between the largest and smallest branches, however, more and more subtle differences in *α* are both discernible and measurable from the total amount of detail in the figure.

### Fractal geometry of *α*

Inquiry into the aesthetics of fractals since its inception has focused on the fractal dimension *D* (7, 13, 14, 17, 19, 34). Fractal dimension generalizes the familiar concept of dimension—called Euclidean or topological dimension—to noninteger values by defining the dimension of a shape as the exponent *D* in a scaling relationship


(5) $$N={\left(\frac{1}{r}\right)}^{D}$$

between the number of units *N* required to completely cover the shape (by overlapping) versus the width of the units *r* (26). For example, a cube is three-dimensional because however many cubes *N* of whatever width *r* it takes to completely overlap a cube, it requires 8 times as many cubes of half the width, so $8N=(1/\frac{r}{2}{)}^{D}$. Dividing this expression again by Eq. 5 gives $8=2^{D}$ and hence D=3. Many alternative ways to define dimension that follow this basic idea produce equivalent dimensions *D*, such as Hausdorff dimension (35) and box-counting dimension (6). Studies of fractals in flat media such as Pollock drip paintings (14), calligraphy (10), and Mondrian tree paintings (19) have measured fractal dimension by decomposing images into black and white shapes and counting the number of boxes of various size required to cover the figure (10, 17, 19).

**Fig. 3.**
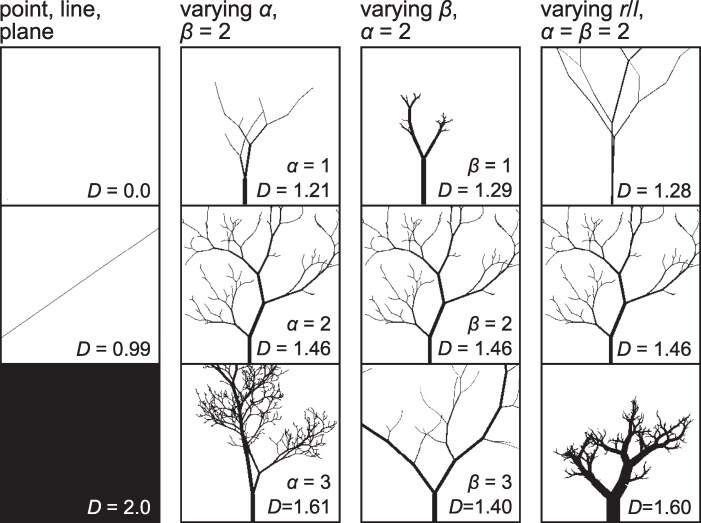
The fractal dimension D generalizes the familiar, topological dimension to noninteger values. Points, lines and planes have the familiar dimensions of 0, 1, or 2 (column 1), whereas tree shapes have noninteger dimension between 1 and 2 (columns 2–4). *D* increases with *α* for the three trees from Fig. 2 (column 2), but also depends on the length scaling exponent *β* (column 3), as well as initial ratio of radius to length of branches (column 4). *D* values shown here are the box-counting dimension of 256×256 binary images computed by regression as the slope of the log of Eq. 5, $\log_{2}\;N∼D\log_{2}\;(1/r)$, the $\log_{2}$ number of occupied squares versus the $\log_{2}$ number of squares across the width of the image.

The radius scaling exponent *α* shares some aspects with the fractal dimension *D*. Both are scaling exponents and both describe how much more detail is visible with increasing magnification. However, *α* depends only on branch diameter whereas *D* depends on the complete figure including branch lengths and other rendering details. In simple figures, as shown in Fig. 3, *D* increases with *α*, all else being equal, but comparable variation in *D* can be achieved by varying parameters of branch lengths such as an analogous length scaling exponent *β* (specifying lengths as $l_{a}^{\beta }+l_{b}^{\beta }=l_{c}^{\beta }$) or the initial ratio of branch radius to length.

Measurements of *α* isolate the effects of proportions among branch thickness and the number of visible branches, whereas measurements of *D* from figures depend on many other factors such as overlap of branches, the presence or the absence of leaves or ornament, and arbitrary choices involved in rendering an image to black and white (14). For example, the fractal dimension of a tree considered as a volume in three-dimensional space is not the same as the fractal dimension of its projection onto a plane. By contrast, *α* is consistent across paintings in different styles, sculpture, and living trees.

### Scaling of trees in art

We analyzed several famous artworks depicting trees to measure and explain *α*. We chose, in a haphazard way, pleasing works in the public domain that depict single trees, with crisp branch diameters, that show large contrast in size between the smallest and largest branches. We chose works spanning cultures and time period to reduce “WEIRD” biases (36). We nonetheless chose works that we find beautiful and we neglect many worthy works.

We measure *α* by hand-annotating limbs after each branch point in digitized artwork, then using common software to extract their diameters (Materials and methods). The radius scaling relationship in Eq. 4 implies that the diameter of a randomly selected branch follows a power law probability distribution (37). We thereby estimate *α* from bulk diameter measurements using maximum likelihood (38) in the form of modern statistics developed for trees and vasculature (29, 39) (Materials and methods). Just as differences in *α* are much more readily discernible in Fig. 2a than Fig. 2b, likewise statistics based on counting the number of branches of different diameters are much more robust estimators of *α* than methods that attempt to solve Eq. 3 at branch points, despite widespread historical use of the later (40, 41). Annotating branch diameters without recording their topology also enables us to score works of art or photographs rapidly compared with measuring living trees (5).

How well the data conform to the power law is revealed in log–log plots of branch diameter *d* versus the percentage of branches that exceed *d*. If the branch diameters are self-similar, these plots yield approximately straight lines with slope equal to −α (see Materials and methods, (29)). Systematic deviation from self-similarity creates overall curvature, while wiggles and jaggedness indicate random variation or measurement error (39). Curvature at small values of *d* indicates a scale at which self-similarity breaks down due to effects at the smallest scale, such as the thickness of the brush. Values subject to small-scale effects are universally thrown away in power law statistics (38, 42). Each plot then provides a compact but comprehensive picture of how well the power law model fits the branch diameter measurements.

**Fig. 4.**
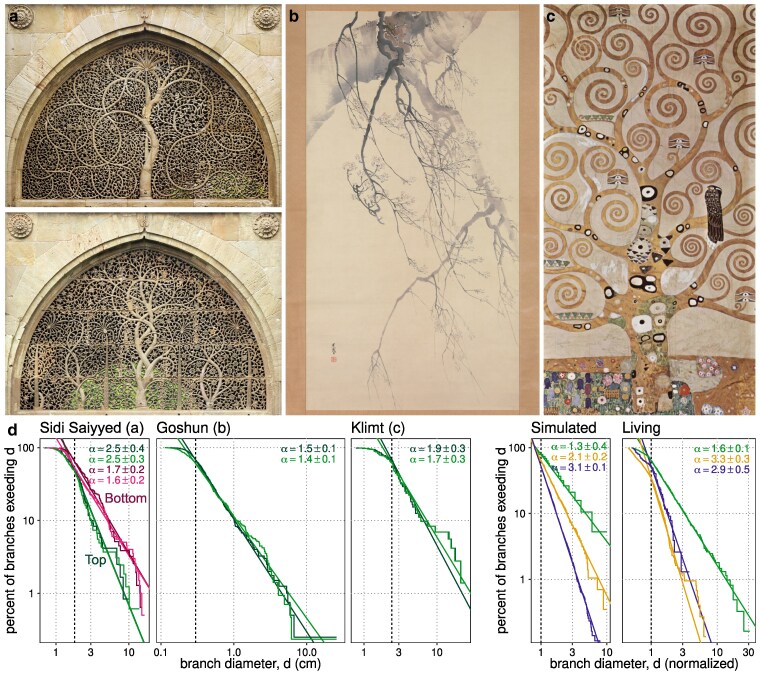
Trees in artwork across centuries, cultures, and styles exhibit fractal scaling. a) carved stone window screen of Sidi Saiyyed Mosque, Ahmedabad, Gujarat, India (1573 CE). b) *Cherry Blossoms*, Matsumura Goshun (1752–1811), ink on paper. c) *L’Arbre de Vie* (Tree of life), Gustav Klimt (1909), oil on canvas. d) data and inference of α±95% CI in two replicate measurements for each work along with simulated (Fig. 2) and living (5) trees. Dotted vertical lines indicate the cutoff for excluding small-scale effects. Diameters from simulated and living trees are divided by this minimum value and therefore the horizontal axis is unitless.

Medieval Islamic design and architecture is known for precise and sophisticated mathematical patterns (43). The late-medieval Sidi Saiyyed Mosque pictured in Fig. 4a features iconic tree patterns carved into stone window screens (jalis). In one (Fig. 4a, top), a single spiraling tree fills the frame, while the second design (bottom) partitions the space among five smaller trees. Both exhibit roughly self-similar radius scaling (Fig. 4d). In the first, conformity to self-similarity is comparable to a dataset of living ponderosa, piñon and balsa trees sacrificed and measured for science (5, 30). Its inferred *α* is 2.5±0.4, between da Vinci’s and Murray’s exponents. Estimates for the second jali fall within the range 1.6–1.7±0.2, even when we do or do not include the smaller four of its five trees. The second figure departs further from self-similarity, however, with a deficit of small branches. This deviation from the model results in underestimation of the exponent, but a reliable upper limit of 2.2 can still be derived from the data and Eq. 4. Differences in the exponent and model fit between the jalis can be attributed to the composition. The second jali does not have room for enough smaller branches, given that the main trunks are mostly of comparable size.

*Cherry Blossoms* is an ink on paper painting by Matsumura Goshun (1752–1811) of Edo period Japan. The diameters of Goshun’s tree conform to self-similar proportions comparably well to living trees and particularly well among the most intricate branches between 3–10 mm. Though extremely rich in detail, with over 400 individual branches, the painting exhibits a scaling exponent of only 1.4–1.5±0.1, statistically excluding da Vinci’s α=2 (P<0.01). This combination of extreme detail and rapidly decreasing branch diameter is made possible by the 200-fold contrast in diameter between the largest and smallest branches. This contrast in size highlights the delicacy of the smallest branches, while the low value of *α* lends an austerity or emptiness to the composition. This emptiness resonates with multilayered rendering of strong and faint branches as if seen through fog. The value of *α* negotiates a tradeoff between dramatically exaggerated proportions between the largest and smallest branches, versus austerity and perhaps practicality: Achieving α=2 across such a contrast in size would require 4,000 branches.

Gustav Klimt’s *L’Arbre de Vie* (Tree of life) represents a 30-fold size difference between the smallest and largest branches—the least we study—and a highly stylized representation, yet our measurements of the scaling exponent estimates are statistically consistent with α=2, at 1.7–1.9±0.3. The trunk and largest branches are larger than self-similarity would prescribe, causing the statistics to modestly underestimate *α*. *L’Arbre de Vie* is one of seven equally sized panels in the Stoclet Frieze, each containing branching spirals. Yet only this center panel contains self-similar branching. The others repeat a spiral motif at a particular scale consisting of 1–3 cm branches. And so these deviations from self-similarity may nonetheless serve to balance the overall composition. Close conformity to self-similarity occurs only over the 2-fold range between 2.4 and 5 cm, accounting for roughly half of the total number of branches.

### The treeless tree

**Fig. 5.**
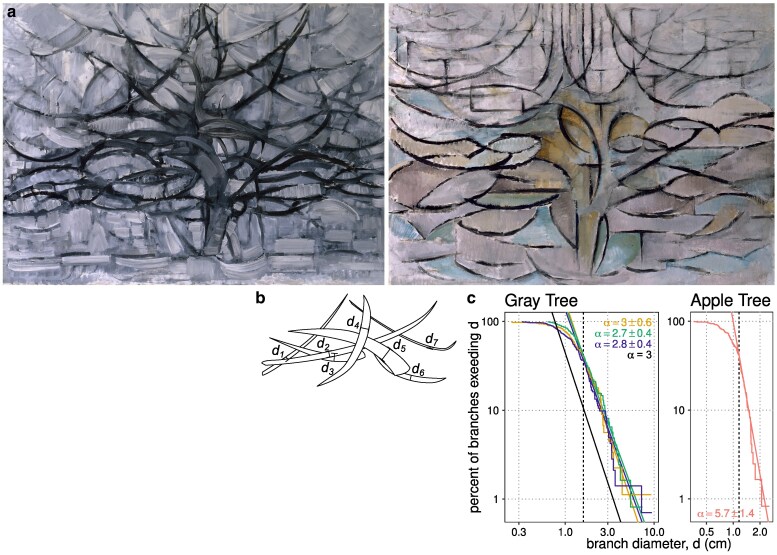
a) *Oil paintings by Piet Mondrian, top: De grijze boom* (*Gray Tree*), 1911, bottom: *Bloeiende appelboom* (*Blooming Apple Tree*), 1912. b) Absent discernible branch points, we visually decompose the figure into arcs and record the approximate diameter of each arc near its visual center of gravity. c) The branch diameter distributions are comparable to living trees for *De grijze boom*, but not for *Bloeiende appelboom*.

Branching is essential to trees. Yet Piet Mondrian—pioneer and theorist of 20th century abstract art famous for the grid-like Mondrian pattern of white and primary-colored rectangles—shows us a tree without branching. Particularly from 1892 to 1912, Mondrian produced many increasingly abstract renderings of trees. These include colorful, realistic and intricately branching trees such as *Laan met roggeschoven* (*Lane with Sheaves of Rye*, c. 1890) as well as trees studied for their fractal aesthetics (19). In the 1911 *De grijze boom* (*Gray Tree*, Fig. 5a top), we see an abstract cluster of dark arcs superimposed at varying angles against a gray field, without obvious branching. The arcs intersect seemingly at random without regard for proportion or relation to adjoining branches, yet from this collection of brushstrokes emerges an unmistakable stark winter tree against a bleak sky.

How does Mondrian communicate this tree so vividly without using the tree’s most essential feature? We argue that *Gray Tree* captures another element just as essential: scaling of branch thickness. Fortunately, our method of measuring *α* applies despite the absence of branching or anatomically relevant points of intersection: if the diameters follow the scaling law in Eq. 4, the result is the same power law we use to measure *α*. We annotate each arc near its visual center of gravity, as in Fig. 5b. Even if different observers choose different center points along similar arcs to measure diameter, this error introduces only an observer-specific constant coefficient *c*, which drops out of Eq. 4. In practice, this process achieves consistency between independent observers (Fig. 5c, Materials and methods).

*Gray Tree* conforms to self-similar scaling of diameter over a 5-fold range of branch diameter with *α* in the range of 2.8−3.0±0.4−0.6 (Fig. 5c, inference ± 95% CI across observers), at the high end of the range of living trees in Fig. 4d. As Mondrian’s tree paintings become increasingly abstract, from colorful, realistic trees with context and foliage such as *Lane with Sheaves of Rye* through the red and blue bare silhouette of *Avond; De rode boom* (*Evening; Red Tree*, 1908), we see the removal of unnecessary elements until in *Gray Tree*, only the most essential features remain. Among these, we still see scaling in branch thickness.

In a subsequent abstract, Mondrian removes even branch scaling, and with it, the effect of a tree. The 1912 *Bloeiende appelboom* (*Blooming Apple Tree*, Fig. 5a bottom) also consists of dark arcs on a gray field. The two paintings could scarcely be more closely matched—in style, date, medium, size, composition, palette, number of arcs, and even the placement of certain arcs that might suggest a trunk or main branches of a particular tree. Yet *Blooming Apple Tree*’s arcs are all around 1 cm in diameter, with only a few exceeding 2-fold larger or smaller. Going through the exercise of measuring scaling produces unreasonable results: a restricted 2.2-fold range of feasibility for the power law and an *α* estimate of 5.7±1.4, well outside the physiologically meaningful range. Likewise, whereas most viewers of *Gray Tree* immediately perceive a tree, naïve viewers of *Blooming Apple Tree* see dancers, roots, fish, faces, water, stained glass, leaves, flowers, or nothing representational at all.

## Discussion and conclusions

We present extensions to a classical perspective on how to appreciate and recreate the beauty of trees, informed by modern tree physiology and fractal geometry. These results can apply directly in the arts, such as refinements to algorithmic tree generation and L-systems (3) as well as refinements to classical guidelines for painting. Our findings highlight how art and science provide complementary lenses on the natural and human worlds. Da Vinci’s prescriptions and the intricate details of the near-contemporaneous Sidi Saiyyed mosque illustrate consilience with a modern scaling theory developed over 400 years later (4, 24), while Mondrian’s test of the limits of visual abstraction results in the same essential features as the mathematical theory.

Trees have become a recurring theme in research on the aesthetics of fractals (11). We provide arguments and evidence for the aesthetic importance of branch diameter scaling and *α* in trees, whereas human subjects research on fractal aesthetics has focused mainly on the fractal dimension *D* (16, 34, 44). We suggest that empirical research on aesthetics could benefit from considering *α*—as a potentially stronger predictor of preference, or to isolate underlying factors involved in fractal dimension or causes of aesthetic preference.

Applications of the theory to larger bodies of work are possible and may yield further insights into art history. Paintings of lightning, akin to trees, became more realistic over time (45) and analysis of composition across thousands of landscape paintings also reveals trends in cultural evolution across art history (46). Such systematic analyzes are now possible for trees. Manual scoring can take as little as 20 min, and automated methods are technically feasible (41).

A classical counter-example to the theory is coppices and pollards. Indeed da Vinci’s description of trees continues, “unless the tree is pollard—if so the rule does not hold good” (1). Pruning a tree at its base or large limbs to produce a recurring supply of small branches was once commonplace in Europe and continues throughout the world. Such coppicing and pollarding breaks self-similar growth, yet pollards have an interesting art history unto themselves and dominate compositions such as Mondrian’s *Willow Grove: Impression of Light and Shadow* or the Song dynasty classic *Along the River During the Qingming Festival*, evoking nostalgia or romantic images of pastoral life. These examples remind us that art may be liked or pleasing for many reasons besides aesthetic proportions, and even in nature, few trees remain perfectly intact (5, 32).

This theory of proportion outlines an ideal, and provides common terms in which to compare art, mathematical models, and the biology of trees. We hope to create dialog across arts, math, and sciences to foster insights into each.

## Materials and methods

### Reproducible research and materials

We chose works in the public domain that depicted trunks or large branches offering sufficient contrast between the largest and smallest branches to measure scaling. Fortuitously, the works are all of comparable size, with the largest boughs between 10 and 26 cm. We selected works to provide a diversity of time period, culture, and style, and we report all works we have ever measured. We decided to measure *Gray Tree* as an out-of-sample validation of the theory after selecting and measuring the first three, then to measure *Blooming Apple Tree* by the same method as a negative control.

### Measuring branch diameter

We produced lists of all branch diameters in each work. We have found in previous work (39) that counting the number of branches at different magnitudes of diameter is a far more accurate measure of *α* than measuring proportions at individual branch points (41). This fact is also evident in the contrast between Fig. 2a and b in terms of sensitivity to *α*. Therefore, we do not even record which diameters correspond to which branches, saving tremendous labor.

We annotate images of each work using lines in the open source scalable vector graphics (SVG) editing program Inkscape. We overlay line segments by hand perpendicular to the direction of the branch to represent each branch diameter. We attempt to choose each diameter as the closest diameter downstream of each branch point that represents the overall branch diameter. That is, we measure diameter downstream of any transient changes in diameter such as the concave curves at Klimt’s branch points or the leaves in Sidi Saiyyed jalis. We then load the SVG file in Mozilla Firefox and use JavaScript code^a^ in the Web Developer Console to extract the branch points. We selected publicly available images that clearly showed the works, avoiding parallax error or shadows that might obscure the branch thickness. We stopped annotating branches when small stems lead only to a single leaf or motif. That is, we do not count as branches the leaves or flowers in Sidi Saiyyed or Goshun or the Egyptian revival decorative motifs in Klimt.

For Mondrian’s trees, which lack branch points, we measure radii by visually decomposing the image into arcs as in Fig. 5b. We score the diameter of each arc without regard to how the arcs intersect and measure the diameter of each arc near its visual “center of gravity,” such as its midpoint, thickest point, or somewhere in between.

As the process is somewhat subjective, each author independently scored each image to control for subjectivity in assessing diameter and the presence or the absence of boughs and branching, resulting in two replicates that show the extent of researcher subjectivity in interpreting the images and scoring rules. Our data files and annotated SVG files are available in the git repository (Data Availability). For Mondrian’s *Gray Tree*, we solicited a third “blinded” replicate from an anonymous participant “a” who was given only an excerpt from this methods section and Fig. 5a (top) and b. For Mondrian’s *Blooming Apple Tree*, we used only one replicate, as no other works yielded statistically significant differences between replicates.

### Statistical estimation

We fit pooled branch diameters to a discrete power-law distribution using a maximum-likelihood method appropriate for branching data (39) following all relevant guidelines in selecting the fit hyperparameters *λ* and $x_{m}$. This method involves logarithmically binning the data according to powers of *λ* to reduce the influence of within-scale noise. We use λ=2, but verify that results do not depend qualitatively on *λ* over a reasonable range (39). Using the discrete power law distribution derived in references (29), the exponent in Eq. 4, interpreted as a probability mass function for radii within each logarithmic bin, is the same as the slope of the log–log tail distribution function in Figs. 4d and 5c. That is, the probability mass function and complimentary cumulative distribution function conveniently have the same exponent. As noted in the reference (29), the exponents of power law probability density functions and cumulative distribution functions differ by 1 due to difference in dimension between probability mass and probability density. We adopt the older convention (following references (29, 39)) of defining *α* as the exponent of the cumulative distribution function, not the density function, which preserves the interpretation of *α* as the exponent of Eqs. 3 and 4.

As usual when fitting power laws (38), we ignore data below a certain threshold, called $x_{m}$ in the literature on power law inference. Data below this threshold are two small to be reliable either due to measurement error or random censorship in the underlying process, such as an artist’s arbitrariness in deciding whether a branch is too small to paint. We chose these thresholds for each work by examining the works themselves and deviations from self-similarity in the data: 1.8 cm for Sidi Saiyyed, 3 mm for Goshun, 2.4 cm for Klimt, and 1.6 cm for *Gray Tree* and 1.2 cm for *Blooming Apple Tree*. The minimum criterion based on the work was that the diameters are reliably measurable. The minimum criterion from the data was that the threshold excluded curvature at low values in the plots in Fig. 4, as this curvature violates the self-similar model and biases the estimation procedure. A quick visual check that the threshold is sufficient is that the slope of the curve matches the slope of the inference at the threshold. This is true for all inferences, as is readily verifiable in Figs. 4d and 5c. Measurements from real trees frequently violate self-similarity, particularly for small branches as bark and other anatomical specifics begin to contaminate the measurements. Hence we chose a separate threshold for each individual tree that produced good fits to the largest branches. In each case, this was also the minimum threshold that passed the visual check.

## Data Availability

All code and research materials for automated reproduction of the analysis are available as a public git repository https://github.com/mnewberry/treescale, including original hand-annotated image files. Hand-annotated image files are available as well in a Data Dryad repository DOI:10.5061/dryad.gb5mkkwxs.

## Footnotes

a
Array.prototype.slice.call(document.getElementsByTagName("path")).map(function(a) { return a.getTotalLength() }).join("∖n");

## Preprints

This manuscript was posted as a preprint, arXiv:2402.13520.
